# Intraoperative Dexmedetomidine Use for Enhanced Recovery after Surgery (ERAS) in Cardiac Surgery—Single Center Retrospective Observational Cohort Study

**DOI:** 10.3390/medicina60071036

**Published:** 2024-06-25

**Authors:** Axel Kerroum, Lorenzo Rosner, Emmanuelle Scala, Matthias Kirsch, Piergiorgio Tozzi, Cécile Courbon, Marco Rusca, Silvijus Abramavičius, Povilas Andrijauskas, Carlo Marcucci, Valentina Rancati

**Affiliations:** 1 Department of Anesthesiology, Lausanne University Hospital, 1005 Lausanne, Switzerlandcarlo.marcucci@chuv.ch (C.M.); valentina.rancati@chuv.ch (V.R.); 2 Department of Cardiac Surgery, Lausanne University Hospital, 1005 Lausanne, Switzerland; 3 Faculty of Biology and Medicine, University of Lausanne, 1015 Lausanne, Switzerland; 4 Department of Intensive Care Medicine, Lausanne University Hospital, 1005 Lausanne, Switzerland; 5 Institute of Physiology and Pharmacology, Lithuanian University of Health Sciences, 44307 Kaunas, Lithuania; 6 II Department of Anaesthesiology and Intensive Care, Vilnius University Hospital Santaros Clinics, 08661 Vilnius, Lithuania

**Keywords:** dexmedetomidine, enhanced recovery after surgery, fast-track anesthesia, early extubation, hospitalization cost

## Abstract

*Background and Objectives*: Dexmedetomidine, an alpha-2 agonist, is used as an adjunct to anesthesia in enhanced recovery after surgery (ERAS) programs. One of its advantages is the opioid-sparing effect which can facilitate early extubation and recovery. When the ERAS cardiac society was set in 2017, our facility was already using the ERAS program, in which the “fast-track Anesthesia” was facilitated by the intraoperative infusion of dexmedetomidine. Our objective is to share our experience and investigate the potential impact of intraoperative dexmedetomidine use as a part of the ERAS program on patient outcomes in elective cardiac surgery. *Materials and Methods*: An observational retrospective cohort study was conducted at a university hospital in Switzerland. The patients who underwent elective cardiac surgery with cardiopulmonary bypass between 1 June 2017 and 31 August 2018 were included in this analysis (*n* = 327). Regardless of the surgery type, all the patients received a standardized fast-track anesthesia protocol inclusive of dexmedetomidine infusion, reduced opioid dose, and parasternal nerve block. The primary outcome was the postoperative time when the criteria for extubation were met. Three groups were identified: group 0—(extubated in the operating room), group < 6 (extubated in less than 6 h), and group > 6 (extubated in >6 h). The secondary outcomes were adverse events, length of stay in ICU and in hospital, and total hospitalization costs. *Results*: Dexmedetomidine was well-tolerated, with no significant adverse events reported. Early extubation was performed in 187 patients (57%). Group 3 had a significantly longer length of stay in the ICU (median: 70 h vs. 25 h) and in hospital (17 vs. 12 days), and consequently higher total hospitalization costs (CHF 62,551 vs. 38,433) compared to the net data from the other two groups (*p* < 0.0001). *Conclusions*: Our findings suggest that dexmedetomidine can be safely used as part of the opioid-sparing anesthesia protocol in patients undergoing elective cardiac surgery with cardiopulmonary bypass with the potential to facilitate early extubation, shorter ICU and hospital stays, and reduced hospitalization costs.

## 1. Introduction

Enhanced recovery after surgery (ERAS) is a multimodal program that reduces postoperative pain and complications induced by the stress of surgery [1]. It has shown its advantages in general surgery, including a reduction in the length of hospital stay and total cost [2,3]. ERAS involves multidisciplinary participation, in which the role of anesthesia remains essential. The ERAS philosophy is increasingly implemented in various surgical disciplines, and the ERAS^®^ cardiac society was created in 2017 [2].

One of ERAS’s pillars is a multimodal, opioid-sparing pain management plan (recommendation class I level B) [4]. This is particularly important in cardiac surgery, where high-dose opioid strategies are associated with adverse effects [5]. Dexmedetomidine is an intravenous selective α-2 receptor agonist that is used for reducing opioid requirements in cardiac surgery patients. Its use for postoperative sedation after cardiac surgery showed significant benefits [6,7,8,9]. However, data are scarce regarding its feasibility for intraoperative administration as part of the ERAS pathway in elective cardiac surgery with cardiopulmonary bypass (CPBV) where early extubation is one of the objectives.

Since 2015, our Swiss University Hospital has been implementing the ERAS pathway in cardiac surgery patients using an opioid-sparing technique with dexmedetomidine as an adjunct to general anesthesia combined with a bilateral multi-level parasternal nerve block.

The aim of this retrospective study was to share our past years’ experience with dexmedetomidine use as an adjunct to anesthesia and its potential impact on patient outcomes following elective cardiac surgery.

## 2. Materials and Methods

### 2.1. Study Design

We performed an observational retrospective cohort study that involved all the adult patients who underwent elective cardiac surgery with CPB and the ERAS pathway during a 14-month period at the University Hospital of Lausanne (Switzerland).

### 2.2. Patient Characteristics

After obtaining approval from the Ethics committee (CER-V n°2019-00167, 21 February 2019), data from 327 adult patients who underwent elective cardiac surgery between 1 June 2017 and 31 August 2018 were collected and analyzed.

### 2.3. ERAS Pathway

The ERAS pathway included standardized anesthesia protocol regardless of the type of surgery and comorbidities:

- No premedication, except if requested by the patient (benzodiazepine).
- A bolus of dexmedetomidine of 0.5 mcg/kg over 10 min before the induction of general anesthesia and immediately followed by a continuous infusion of 0.5 mcg/kg/h.
- Induction of anesthesia: Propofol and sufentanil, with rocuronium or cisatracurium, and:
  o A total of 4 g of intravenous magnesium sulfate before the surgical incision.
  o A bolus of 10 mg/kg of tranexamic acid before surgical incision, followed by a continuous infusion of 1 mg/Kg/h until the end of surgical hemostasis.
- Maintenance of anesthesia:
  o Sevoflurane titrated to a BIS value between 40 and 60 before and after the cardiopulmonary bypass (CPB).
  o Continuous propofol infusion monitored by BIS (value between 40 and 60) during CPB.
  o Top-up doses of sufentanil if needed, up to a total maximum dose of 1 mcg/Kg.
  o The dexmedetomidine infusion is stopped after weaning from cardiopulmonary bypass.
- Parasternal nerve blocks:

Bilateral multi-level parasternal blocks were performed by the surgeon using 40 mL of bupivacaine 5% with epinephrine 1/200,000 at the end of the surgery.

- Fluid management:

Restrictive fluid therapy, guided by hemodynamic monitoring to avoid volume overload. Transesophageal echocardiography is the preferred technique for fluid guidance, performed by European certified cardiac anesthesiologists.

- Patient blood management:

The systematic intraoperative use of antifibrinolytics and cell salvage, and permissive transfusion targets, i.e., hemoglobin concentration < 70–80 g/L according to clinical judgment.

### 2.4. Extubation and ICU Admission

All the patients were considered candidates for extubation in the operating room irrespective of comorbidities and the type of cardiac surgery with the use of CPB. Specifically, the patients with severe contractile dysfunctions, pre-existing respiratory conditions, or combined surgeries were not excluded. The decision to extubate the patient at the end of the surgery in the operating room was made by the responsible cardiac anesthesiologist based on hemodynamic stability, the absence of significant bleeding, metabolic or respiratory acidosis, the presence of an adequate neurological state, proper oxygenation, and well-controlled pain.

All the patients were transferred to the intensive care unit. Non-invasive mechanical ventilation commenced and continued in the ICU for two hours for the extubated patients.

Other non-medical reasons could be the cause of the delay in extubation such as logistical issues involving the unavailability of a respiratory therapist or time constraints.

### 2.5. ICU Discharge Criteria

The patients were discharged from the ICU if they had hemodynamic stability without the need for continuous intravenous medications, except for very low norepinephrine doses; respiratory stability and satisfactory oxygenation levels (normal for the patient); and normal neurological status, and they were awake and alert without significant neurological deficits, with a well-controlled pain with oral medications.

### 2.6. Data Collection

Demographic, intraoperative, and postoperative data were retrospectively retrieved from the institute’s administrative database AXYA (Cerner France, Puteaux, France) the electronic patient files Soarian (Cerner corp. North Kansas City, MO, USA) and Metavision 5 (IMDsoft, Tel Aviv, Israel), and the anesthesia record.

The following variables were collected:

#### 2.6.1. Preoperative

- General: age, sex, BMI, comorbidities, treatment, cardiovascular risk factors, and ASA classification.
- Clinical and Biological: hemoglobin, renal function, left ventricular ejection fraction (LVEF), and NYHA class.

#### 2.6.2. Intraoperative

- The duration of the procedure, duration of CBP, duration of aorta cross-clamping, and nadir temperature during CBP.
- Blood loss and transfusion requirements.
- Opioids administered and the duration of dexmedetomidine infusion.
- Hypotension (<55 mmHg mean arterial pressure) and hypertension (BP > 25% above the baseline).
- Bradycardia (<50 beats per minute (bpm)) and tachycardia (HR > 25% above the baseline).
- Insulin requirement (insulin drip started if blood glucose > 10 mmol/L).
- Inotropic or vasopressor support.
- Sternal infiltration by local anesthetics (mL).

#### 2.6.3. Postoperative

- Extubation time.
- Blood loss and transfusion requirement within the first 24 h.
- If reintubation is needed (for patients extubated in the OR).
- If surgical re-exploration is needed.
- Postoperative complications: cardiac, renal, neurological, infectious, and respiratory.
- Postoperative opioid consumption calculated in Morphine Milligram Equivalent.
  (MME = iv morphine (mg) + iv fentanyl (mcg)/10 + oral oxycodone (mg)/2)
- Length of stay in ICU (ICU LOS).
- Length of stay in hospital (HOSP LOS).
- Mortality at 30 days.
- Total hospitalization costs.

#### 2.6.4. Endpoints

Intraoperative safety endpoints: the incidence of hypotension, hypertension, hyperglycemia, cardiac arrythmias, and need for vasoactive drugs. Postoperative safety endpoints: the incidence of postoperative complications, time of extubation, ICU and hospital LOS, and total cost of hospitalization.

### 2.7. Statistical Analysis

The distribution of data was evaluated using the Kolmogorov–Smirnov test. Normal distributed data are represented as mean (±SD, range), and non-normal distributed data are represented as median (IQR, range). Frequencies are represented as numbers (%).

Three groups were identified based on mechanical ventilation time: group 0: extubated in the OR, group < 6: extubated in the ICU within 6 h postoperatively, and group > 6: extubated in the ICU after 6 h postoperatively.

Group comparisons were made using the Wilcoxon rank sum and Kruskal–Wallis tests for continuous variables and Pearson’s chi 2 for continuous variables. *p* < 0.05 was considered statistically significant after correction for multiple comparisons using the Bonferroni method. Statistical analysis was performed using the JMP 15 software.

## 3. Results

### 3.1. Preoperative Variables

The authors collected data from 327 consecutive adult patients who underwent elective cardiac surgery with cardiopulmonary bypass (CPB). A total of 83 (25.4%) of the patients were female. The median age was 66 years (IQR 57–73, range 20–80), and the median BMI was 29.5 kg/m^2^ (IQR 23.6–29.6, range 17.8–48.6). The most common comorbidities were hypertension, coronary artery disease, and chronic kidney disease. The prevalence of comorbidities, medication use, ASA status, NYHA class, and chronic kidney disease (CKD) class are summarized in Table 1.

### 3.2. Intraoperative Variables

The most common interventions were coronary artery bypass grafting (CABG) (30.3%), valve repair or replacement (25.7%), and major aorta surgery (aortic root and ascending aorta) (27.2%). The median duration of surgery, CPB, and cross-clamp time were 307 min, 84 min, and 60 min, respectively (Table 2).

The median nadir temperature was 35.9 °C (IQR 35.5 to 36.2, range 27.4 to 36.7) and the median last recorded temperature before transport to the ICU was 36.5 °C (IQR 36.1 to 36.8, range 34–38).

The median total dose of sufentanil administered during surgery was 0.65 mcg/kg (IQR 0.5–to 0.8, range 0.2 to 1.6), and the mean dexmedetomidine perfusion time was 240 min (IQR 180 to 300, range 0 to 480).

Hypotension occurred in 13.6% of the patients and required a vasopressor treatment with phenylephrine boli and norepinephrine infusion. Hypertension was present in 20 patients (6.2%) and was controlled by adjusting analgesia and the depth of anesthesia when needed, or with vasodilators (nitroglycerine or nicardipine boli). Bradycardia occurred in 81 patients (25.1%), of whom the dexmedetomidine was interrupted in 20 patients (6%), and anticholinergic agents (atropine or glycopyrrolate) were given in 29 patients (9%). Hyperglycemia requiring insulin (>10 mmol/L) occurred in 92 patients (28.5%).

After weaning from CPB, 98% of the patients were treated with norepinephrine, 19% received dobutamine, 20% received epinephrine, and 4% received milrinone. The highest infusion doses of vasopressors and inotropes are presented in Table 3.

### 3.3. Emergence and Extubation

The distribution of the patients according to their age, BMI, disease stage, and type of surgery in the three groups of postoperative mechanical ventilation duration is shown in Table 4.

A total of 187 patients (57%) were extubated in the OR at the end of the surgery. Of the remaining 140 patients, 70 (21.5%) were extubated within 6 h after arrival in the ICU, and 70 (21.5%) were extubated more than 6 h after arrival in the ICU (Table 5).

None of the patients extubated in the OR were reintubated after arrival in the ICU.

The reasons not to extubate the patient in the OR included the presence of metabolic acidosis (26.9%), hemodynamic instability (22.6%), ongoing bleeding (14%), low FiO_2_/PaO_2_ ratio (8.8%), hypothermia (0.7%), and logistical reasons (30.5%) No reason for delayed extubation could be found in the records of 29.4% of the patients (Table 6).

### 3.4. Postoperative Course

Postoperative opiate consumption expressed in MME was similar in the group extubated in the OR with 28 mg (IQR 15.5–51, range 0–204) and the group extubated within 6 h with 30 mg (IQR 19.5–59.3, range 4–219) (*p* = 0.24). In the group extubated after 6 h, it was significantly increased compared to both the other groups with 70 mg (IQR 46.3–107.5, range 7.5–245) (*p* < 0.0001)**.** The most common complications were second or third-degree atrioventricular block in 26 patients (8.2%). In total, 17 patients (5.4%) required a permanent pacemaker (Table 7). Postoperative vasoplegia was present in 18 patients (5.7%). In total, 21 patients (6.7%) suffered postoperative delirium (CAM-ICU: the Confusion Assessment Method used in ICU). Acute kidney injury was diagnosed in 32 patients (10.1%) of whom 5 (1.6%) required renal replacement therapy.

### 3.5. Length of Stay (LOS) in ICU, LOS in Hospital, and Cost

**Figure 1:**
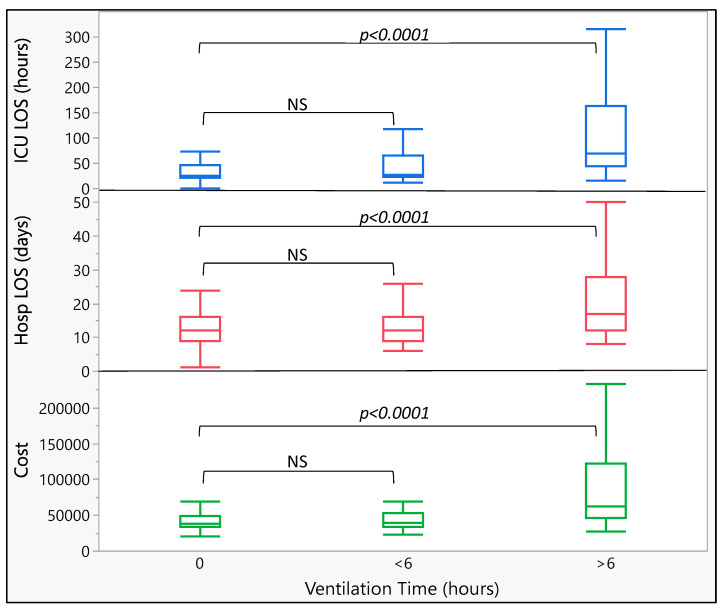
Comparison of LOS and cost between extubation in OR, extubation within 6 h postop, and extubation after 6 h postop. Ventilation time: 0: extubation in OR at end of surgery, <6: extubation within 6 h of end of surgery, and >6: extubation later than 6 h after end of surgery. CHF: Swiss Franc, Boxes represent median and IQR and whiskers represent 5th and 95th percentile. NS: non-significant.

ICU LOS, Hospital LOS, and total hospitalization cost are presented in Figure 1. There was no difference between the patients extubated in the OR and those extubated within 6 h postoperatively. All three parameters were significantly increased for the patients extubated later than 6 h postoperatively. The significance level of the difference (*p* < 0.0001) between the groups remained the same after the exclusion of the two outliers in the group of the patients with ventilation times longer than 6 h (ICU LOS 1454 and 1844 h, Hosp LOS 152 and 155 days and cost CHF 793’438 and 886’255).

## 4. Discussion

The advantages of using dexmedetomidine in perioperative cardiac surgical care are well established, but almost all the studies concentrate on its use as a sedative in the postoperative period, or on postoperative outcomes. Limited data are published on the intraoperative effects of dexmedetomidine as an adjunct to general anesthesia.

In two meta-analyses comprising 52 studies [10,11], only 23 studies reported on the intraoperative use of dexmedetomidine. In the remaining 29, dexmedetomidine was administered only for postoperative sedation in the ICU.

A total of 18 of the 23 studies, where dexmedetomidine was used intraoperatively, only had postoperative outcome measures such as the markers of myocardial injury, renal injury, inflammation, conduction block, and cognitive function.

Of the remaining five, two used dexmedetomidine only as a bolus at the induction of anesthesia to blunt the sympathetic response to endotracheal intubation, and two concerned off-pump cardiac surgery. Only one study dating from 1997 [12] describes its use as an adjunct to general anesthesia in cardiac surgery. In this randomized, double-blind trial, 40 patients received a placebo and 40 received dexmedetomidine at 0.5 mcg/Kg/min for 30 min prior to the induction of anesthesia and 0.07 mcg/Kg/min from the induction to the end of surgery. Further, high dose opioid (fentanyl 30 mcg/Kg) was used for induction and maintenance (0.15 mcg/Kg/min), and completed with enflurane inhalation anesthesia. Their study population was highly selective to exclude severe cardiac (ventricular dysfunction, severe valvular dysfunction, and > 50% left main stem stenosis) and other systemic illnesses.

They found that dexmedetomidine use resulted in lower blood pressure and heart rate at induction, during intubation, and throughout surgery. The incidence of hypotension, defined as a drop of >30% from baseline, was similar in both the groups, yet systolic blood pressure drop below 90 mmHg was more frequent in the treatment group. Tachycardia was more frequent in the placebo group and bradycardia was similar, with eight patients (20%) in each group requiring pharmacological intervention. Post CPB pacing for AV conduction block was more frequent in the treatment group, but the difference did not reach statistical significance.

In a much more recent trial, Aguerreche et al. [13] retrospectively compared 40 patients in whom an opioid-free anesthetic protocol was used during cardiac surgery with 40 patients who were treated with an opioid-based anesthetic regimen. The opioid-free strategy comprised dexmedetomidine (bolus and continuous infusion), magnesium sulfate, dexamethasone, lidocaine (bolus and continuous infusion), and ketamine (bolus and continuous infusion). In the opioid-based strategy, remifentanil and propofol were used as target-controlled infusions. The comparison focused on postoperative opioid consumption and pain scores. They also compared intraoperative hemodynamic variables and postoperative complications. In terms of opioid reduction, through the use of Dexmedetomidine intraoperatively, Coustrouglo et al. reached the same results [14].

The dexmedetomidine group required less morphine in the first 24 h after surgery (15 mg vs. 30 mg) while having lower pain scores (significant only during coughing). They did not find a difference in the incidence of bradycardia, nor in the intraoperative use of norepinephrine; yet, more patients in the opioid-based group required ephedrine boli.

Hemodynamic changes that are reportedly associated with dexmedetomidine infusion are hypertension, hypotension and bradycardia, atrial fibrillation, and cardiac conduction blocks.

We evaluated the effect of dexmedetomidine on blood pressure and heart rate during the pre-CPB phase of surgery, since bypass, bleeding, and the surgical procedure itself are major confounding factors. Hypertension and hypotension after the onset of the dexmedetomidine infusion occurred in 6.2% and 13.6% of the patients, respectively, but were easily manageable in all the cases using short-acting vasodilators or vasopressors. Bradycardia is a more frequent side effect (25% of the patients) easily managed by the administration of anticholinergic drugs in 8.6% or even the interruption of the infusion in 6% of the patients.

Postoperative atrial fibrillation was more frequent in the patients undergoing valve surgery (32.1%) than in the patients undergoing CABG surgery (24.5%). These incidences are consistent with the reported data [15,16,17] and indicate that the intraoperative use of dexmedetomidine is unlikely to influence the occurrence of atrial fibrillation. Atrioventricular second- and third-degree conduction blocks were much more frequent in surgical procedures including valve repair or replacement than in isolated CABG, requiring permanent pacing in 5.4% of the cases. Again, these results are consistent with the reported data [18], and suggest that dexmedetomidine does not increase the risk of permanent high-grade conduction block.

Taking into consideration the analgesic effect of dexmedetomidine, opioid use was minimal. An opioid-sparing technique is an essential prerequisite for early extubation.

Only 39 patients (12%) received more than 1 mcg/Kg of sufentanil during surgery, and the maximum dose administered was 1.6 mcg/Kg. However, due to its relatively short elimination half-life, the intraoperative administration of dexmedetomidine does not provide postoperative analgesia. The median 24 h Morphine Milligram Equivalent (MME) consumption in our subgroups of patients who were extubated immediately or within 6 h were 28 mg (IQR 15.5–51) and 30 mg (IQR 19.5–59.25), respectively. These results are similar to the results reported by Barr et al. [19] who compared bilateral multi-level parasternal blocks using ropivacaine 0.75% with a placebo. They found reduced pain scores and opioid consumption in the treatment group which had a 24 h MME consumption of 30.8 mg, identical to our results. This suggests that the addition of dexmedetomidine during surgery does not reduce opioid consumption beyond its intraoperative effect.

Postoperative delirium is one of the most frequent complications after cardiac surgery [20], and has been a research topic for improvement for decades. While many studies describe a significant reduction in delirium when using dexmedetomidine in the ICU [21], this has been questioned based on the results of some recent trials [22,23,24]. We diagnosed a very low number of patients (6.7%) suffering from postoperative delirium (CAM-ICU score >3), compared to the published rates going from 17 to 24% in the patients receiving dexmedetomidine. This discrepancy is likely due to the different definitions of delirium used. Most of our patients (57.2%) were extubated in the operating room (OR) after surgery, but logistical factors were the most common reason for non-extubation. In fact, the implementation of non-invasive mechanical ventilation (NIV) following surgery has effectively mitigated respiratory complications such as atelectasis and CO_2_ retention. However, the restricted availability of NIV to anesthetists beyond 5 pm at our center introduces a potential confounding factor in our dataset. This limitation could obscure any advantages associated with early extubation, as no significant differences in the ICU length of stay (LOS) or cost were observed between the patients extubated in the operating room or shortly after ICU admission.

Additionally, our hospital discharges patients from the ICU in the morning, even if they meet the criteria for transfer to the ward the night before. Also, our stepdown unit cannot accommodate cardiac surgery patients immediately after surgery. While several of the patients extubated in the OR probably did not require ICU surveillance, the mandatory transit through the ICU increases their hospitalization cost and masks any benefits of early extubation.

This retrospective study has many limitations. The most obvious is the absence of a control group, making it difficult to determine if the observed outcomes are due to the intervention or other confounding factors. As for all the retrospective analysis, data collection relies on existing records, which may be incomplete or biased. The quality of the data might have affected the results as much as the intervention studied. The studied population includes patients with very diverse characteristics, requiring caution when generalizing the results to specific subgroups and contexts.

## 5. Conclusions

This study provides several clinically useful conclusions regarding the feasibility of using intraoperatively dexmedetomidine in this patient population. The results suggest that it can be used safely and effectively with opioid-sparing anesthesia to achieve an early extubation in patients undergoing cardiac surgery. Its use is associated with the expected side effects of bradycardia, hyperglycemia due to hyperinsulinemia, and hypotension but only to a limited degree which can easily be managed. Consequently, a short duration of stay in the ICU and in the hospital and a reduced cost of hospitalization can be achieved.

Our study encourages further research to confirm our findings and determine the optimal dose of dexmedetomidine administration.

## Figures and Tables

**Table 1:** Demographics and preoperative variables.

		N (%)	Median (IQR)
Age (years)		327	66 (57–73)
Gender	F	83 (25.4%)	
	M	244 (74.6%)	
ASA	1	1 (0.3%)	
	2	22 (6.7%)	
	3	245 (74.9%)	
	4	59 (18.0%)	
BMI (kg/m^2^)		327	25.9 (23.6–29.7)
Smoking		112 (34.3%)	
Alcohol		75 (22.9%)	
Diabetes		59 (18.0%)	
High BP		217 (66.4%)	
Hyperlipidemia		174 (53.2%)	
Ischemic heart disease		183 (56.0%)	
Arrhythmia		68 (20.8%)	
COPD		35 (10.7%)	
Periph. vasc. disease		47 (14.4%)	
NYHA class	I	22 (6.7%)	
	II	190 (58.1%)	
	III	104 (31.8%)	
	IV	11 (3.4%)	
Calcium blockers		53 (16.4%)	
Beta blockers		145 (44.9%)	
ACEI/ARB		176 (54.5%)	
Antiarrhythmics		20 (6.2%)	
Statins		150 (46.4%)	
Aspirin		164 (51.1%)	
Anti P2Y12 stopped < 5 d		18 (5.6%)	
DOAC stopped < 24 h		10 (3.1%)	
AVK stopped < 24 h		7 (2.2%)	
Preoperative LVEF (%)		327	60 (50–65)
LVEF < 40%		40 (12.2%)	
Preoperative Hb (g/L)		327	138 (125–148)
Preoperative eGFR		327	60 (60–60)
CKD class	1	261 (79.8%)	
	2	37 (11.3%)	
	3	25 (7.6%)	
	4	3 (0.9%)	
	5	1 (0.3%)	

ASA: American Society of Anesthesiologists, BMI: body mass index, BP: blood pressure, COPD: chronic obstructive pulmonary disease, NYHA: New York Heart Association, ACEI: Angiotensin-converting enzyme inhibitors, ARB: Angiotensin receptor blockers, DOAC: direct oral anticoagulants, AVK: vitamin K antagonists, LVEF: left ventricle ejection fraction, Hb: hemoglobin, eGFR: estimated glomerular filtration rate, and CKD: chronic kidney disease.

**Table 2:** Intraoperative variable.

	N (%)	Median (IQR)
Type of Surgery:		
CABG	99 (30.3%)	
CABG + Valve	33 (10.1%)	
CABG + Double Valve	1 (0.3%)	
Aortic Root & Ascending Aorta	89 (27.2%)	
Valve	84 (25.7%)	
Double Valve	19 (5.8%)	
Aortic Arch	2 (0.6%)	
Duration of Surgery (min)		307 (259–365)
Duration of CPB (min)		84 (63–114)
Cross Clamp Time (min)		60 (45–88)
Temperature Nadir (°C)		35.9 (35.5–36.2)
Last Temp. (°C)		36.5 (36.1–36.8)
Sternal Infiltration (ml)	118 (38.0%)	40 (30–40)
Bleeding and Transfusion:		
Cell-Saver (ml treated)		650 (474–800)
RBC Units (intraoperative)	28 (9.0%)	2 (1–2.75)
IABP	2 (0.6%)	
ECMO	8 (2.5%)	
Re-intervention.	43 (13.2%)	

CABG: coronary artery bypass graft, CPB: cardiopulmonary bypass, RBC: red blood cell, IABP: intra-aortic balloon pump, ECMO: extracorporeal membrane oxygenation, and Re-intervention: the return of the patient to the operating room after admission to the ICU for a surgical reason (bleeding, tamponade, infection, etc.).

**Table 3:** Intraoperative use of dexmedetomidine.

	N (%)	Median (IQR)
Side Effect		
Hypotension	44 (13.6%)	
Hypertension	20 (6.2%)	
Bradycardia	81 (25.1%)	
Hyperglycemia *	92 (28.5%)	
Intervention		
Norepinephrine	316 (98%)	
Atropine	1 (0.3%)	
Glycopyrrolate	28 (8.6%)	
Dexmedetomidine Interruption	20 (6.%)	
Sufentanil Doses (mcg/kg)		0.65 (0.50–0.80)
Dexmedetomidine Infusion Time (min)		240 (180–300)
Inotropic and Vasopressor Support		
Norepinephrine (mcg/kg/min)	315 (98.0%)	0.062 (0.041–0.094)
Dobutamine (mcg/kg/min)	62 (19.0%)	3.5 (2.8–4.5)
Epinephrine (mcg/kg/min)	64 (20.0%)	0.039 (0.027–0.059)
Milrinone (mcg/kg/min)	13 (4.0%)	0.339 (0.301–0.496)
Insulin	92 (28.5%)	

* Hyperglycemia: dexmedetomidine’s side effect, described in the Swiss compendium as frequent.

**Table 4:** Ventilation time by type of surgery, BMI, age, NHYA, and LVEF.

Ventilation Time	0	>6 h	<6 h
Type of Surgery			
CABG	66	20	13 *
CABG + Valve	15	4	14
CABG + Double Valve	0	0	1
Aortic Root & Ascending Aorta	44	22	23
Valve	52	17	15
Double Valve	9	7	3
Aortic Arch	1	0	1
BMI (mean ± SD)	26.3 (±4.2)	26.9 (±4.5)	28.1 (±6)
Age (mean ± SD)	63.6 (±13.5)	64.5 (±13.8)	65.4 (±13.4)
NYHA class			
I	17	4	1
II	113	41	36
III	52	23	29
IV	5	2	4
LVEF < 40%	13	11	16 *

CABG: coronary artery bypass graft, BMI: body mass index, NYHA: New York Heart Association, and LVEF: left ventricle ejection fraction. * type of surgery and LVEF <40% were significantly different between groups 0 and >6 h (*p* < 0.0125).

**Table 5:** Extubation time.

	N	%
Extubated in OR at the end of surgery		
No	140	42.8%
Yes	187	57.2%
ICU ventilation time		
0	187	57%
<6 h	70	21.5%
>6 h	70	21.5%
Reintubation in ICU after extubation in OR	0	

OR: operating room, ICU: intensive care unit.

**Table 6:** Reasons for non-extubation with group comparison.

Reasons for Non-Extubation in OR	<6 h	>6 h	
	n (%)	n (%)	P
Logistical reasons			
End after 5 pm *	16 (23)	17 (24)	0.8418
First of the list **	4 (5)	5 (7)	0.7301
Unknown Reasons	27 (39)	13 (19)	0.0084
Metabolic acidosis	10 (15)	26 (38)	0.0026
Hemodynamic instability	10 (14)	21 (30)	0.0278
Bleeding	3 (4)	16 (23)	0.0013
Low FiO_2_/PaO_2_	3 (4)	9 (13)	0.0782
Hypothermia	1 (1)	0 (0)	0.3120
Other	6 (8)	10 (15)	0.2871

OR: operating room; unknown reasons: non-logistic and non-medical but undocumented; * end after 5 pm: the elective program ends at 5 pm in our hospital, involving a reduction in staff afterwards; ** first of the list: due to time constraint, and the preparation of the operating room for the continuation of the list.

**Table 7:** Postoperative complications by group.

	0	<6 h	>6 h	*p*
	n (%)	n (%)	n (%)	
Cardiac				
STEMI/NSTEMI	4 (2.20%)	2 (3.03%)	0 (0.00%)	0.395
AVB I	9 (4.95%)	5 (7.58%)	4 (5.88%)	0.729
AVB II or III	12 (6.59%)	8 (12.12%)	6 (8.82%)	0.368
BBB	7 (3.85%)	6 (9.09%)	5 (7.35%)	0.232
Afib/Aflut	56 (30.77%)	24 (36.36%)	24 (35.29%)	0.635
SVA	4 (2.20%)	3 (4.55%)	4 (5.88%)	0.319
VA	4 (2.20%)	4 (6.06%)	0 (0.00%)	0.075
Tamponade	6 (3.30%)	4 (6.06%)	7 (10.29%)	0.08
Vasoplegia	5 (2.75%)	3 (4.55%)	10 (14.71%)	0.001 *
Def pacemaker	10 (5.49%)	4 (6.06%)	3 (4.41%)	0.909
Respiratory				
Pneumonia	7 (3.85%)	2 (3.03%)	10 (14.71%)	0.003 *
Pulmonary edema	6 (3.30%)	2 (3.03%)	4 (5.88%)	0.594
Respiratory failure	21 (11.54%)	9 (13.64%)	7 (10.29%)	0.829
Renal				
ARF	13 (7.14%)	6 (9.09%)	13 (19.12%)	0.019 *
Dialysis	2 (1.10%)	2 (3.03%)	1 (1.47%)	0.561
Neuro				
Confusion/delirium	7 (3.85%)	5 (7.58%)	9 (13.24%)	0.028 *
Stroke	2 (1.10%)	1 (1.52%)	8 (11.76%)	0.0001 *^#^
Infection	20 (10.99%)	2 (3.03%)	11 (16.18%)	0.0423 ^#^
Other complications	41 (22.53%)	14 (21.21%)	15 (22.06%)	0.976
Mortality				
Cardiac death	0 (0.00%)	0 (0.00%)	2 (2.99%)	0.024 *
Global 30-day mortality	2 (1.07%)	1 (1.43%)	2 (2.86%)	0.587

Afib: atrial fibrillation, Aflut: atrial flutter, AVB: atrio-ventricular block, BBB: bundle branch block, SVA: supraventricular arrhythmia, VA: ventricular arrhythmia, ARF: acute renal failure. * significant difference between group 0 and group <6 h. # significant difference between group >6 h and group >6 h.

## Data Availability

The datasets used and/or analyzed during the current study are available from the corresponding author upon reasonable request.
